# Diverticules de Meckel compliqués: à propos de 15 cas

**DOI:** 10.11604/pamj.2018.29.81.12675

**Published:** 2018-01-29

**Authors:** Abib Diop, Ousmane Thiam, Mouhamadou Lamine Guèye, Mamadou Seck, Alpha Oumar Touré, Mamadou Cissé, Madieng Dieng

**Affiliations:** 1 Service de Chirurgie Générale, CHU Aristide Le Dantec, Dakar, Sénégal

**Keywords:** Diverticule de Meckel, occlusion, péritonite, Meckel diverticulum, occlusion, peritonitis

## Abstract

Le diverticule de Meckel (DM) est une persistance du canal omphalo-mésentérique. Généralement bénin et asymptomatique, il est surtout découvert fortuitement. Sa découverte se fait souvent à l’occasion de complications. Le but de notre étude est de décrire les aspects épidémiologiques, cliniques, anatomopathologiques et thérapeutiques des complications des DM. Il s’agissait d’une étude rétrospective sur 13 ans (Janvier 2003-Juin 2016) portant sur 15 cas de diverticules de Meckel compliqués pris en charge aux urgences chirurgicales de l’Hôpital Aristide Le Dantec de Dakar. Il s’agissait de 10 hommes et de 5 femmes dont l’âge moyen était de 27,8 ans avec des extrêmes de 1 mois et 73 ans. Les 2 principales circonstances de découverte étaient le syndrome occlusif et l’irritation péritonéale. La laparotomie en urgence avait permis d’affirmer l’implication d’un DM dans le tableau clinique. Dans les cas d’occlusion, le mécanisme était toujours une bride. Dix patients présentaient une nécrose intestinale avec perforation au moment du diagnostic. Tous les 15 patients avaient eu une résection segmentaire du grêle emportant le diverticule. Cette résection était suivie d’une anastomose immédiate dans 12 cas. La morbidité était constituée par 2 cas de fistules et 2 cas de péritonites post opératoires. Un décès par choc septique a été noté. Trois cas d’hétérotopie muqueuse ont été constatés dont une hétérotopie gastrique, une colique et une association d’hétérotopies colique et gastrique chez le même patient. Les complications de DM constituent des urgences digestives nécessitant une prise en charge chirurgicale précoce et adaptée. Cette dernière est greffée d’une morbidité non négligeable.

## Abstract (en)

We conducted a retrospective study of 15 patients with complicated Meckel diverticula treated in the emergency surgery at the Aristide Le Dantec Hospital, Dakar, over a period of 13 years (January 2003-June 2016). The study included 10 men and 5 women, whose average age was 27.8 years, ranging between 1 months and 73 years. The two main circumstances of detection were occlusive syndrome and peritoneal irritation. Emergency laparotomy allowed clinicians to affirm the involvement of Meckel diverticulum in the clinical picture. In the case of occlusion, the mechanism was always a flange. Ten patients had intestinal necrosis with perforation at the time of diagnosis. All 15 patients underwent segmental resection of the intestine with elimination of the diverticulum. This resection was followed by immediate anastomosis in 12 cases. The morbidity was constituted of 2 cases of fistulas and 2 cases of postoperative peritonitis. A case of death due to septic shock was reported. Three patients had heterotopic mucosa, including gastric heterotopia, colic heterotopia and an association between colic heterotopia and gastric heterotopia in the same patient. The complications of Meckel diverticula are digestive emergencies requiring early and adapted surgical treatment. This is characterized by a non-negligible morbidity.

## Introduction

Le diverticule de Meckel (DM) est une persistance du canal omphalo-mésentérique décrit pour la première fois en 1598 [1]. Il s’agit de la malformation congénitale la plus fréquente du tube digestif. Son incidence se situe entre 1 et 4% de la population générale [2]. Généralement bénin et asymptomatique, le DM est une pathologie de l’enfant mais qui peut se manifester à l’âge adulte. Les formes compliquées intéressent 4 à 16% des DM et constituent souvent leurs circonstances de découverte [1-3]. Le but de notre étude est de décrire les aspects épidémiologiques, cliniques, anatomopathologiques et thérapeutiques des complications des DM.

## Méthodes

Nous avons mené une étude rétrospective sur 13 ans (Janvier 2003-Juin 2016) portant sur 15 cas de diverticules de Meckel compliqués pris en charge aux urgences chirurgicales de l’Hôpital Aristide Le Dantec de Dakar. Les paramètres épidémiologiques, cliniques, anatomopathologiques et thérapeutiques ont été étudiés.

## Résultats

Il s’agissait de 10 hommes et de 5 femmes (sex-ratio de 2). L’âge moyen des patients était de 27,8 ans avec des extrêmes de 1 mois et 73 ans. La tranche d’âge de 0 à 30 ans était la plus représentée. Un patient avait un antécédent d’appendicectomie par voie de Mac Burney. Les circonstances de découverte étaient le syndrome occlusif (5 cas) et l’irritation péritonéale (5 cas). Cinq patients présentaient une péritonite occlusive. Le délai moyen de consultation était de 3 jours. La radiographie de l’abdomen sans préparation permettait de confirmer le syndrome occlusif en montrant des niveaux hydro-aériques de type grêlique (10 cas). Un scanner abdominal avait été réalisé chez 1 patient. Il objectivait des images en faveur d’un diverticule de Meckel associé à une dilatation des anses grêles avec une zone transitionnelle de même qu’un épaississement pariétal des anses rehaussé par le produit de contraste. La laparotomie en urgence avait permis d’affirmer l’implication d’un DM dans le tableau clinique.

**Figure 1:**
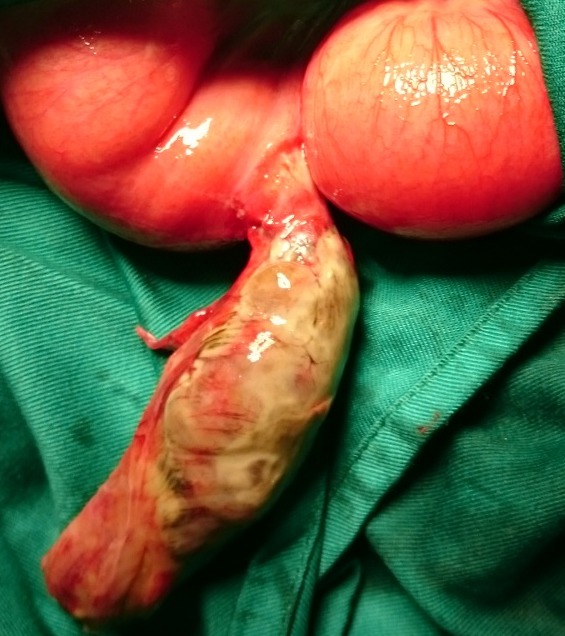
Diverticule de Meckel nécrosé

**Figure 2:**
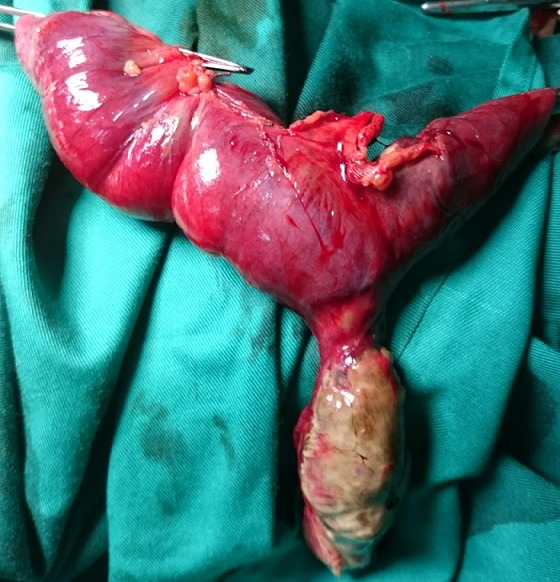
Diverticule de Meckel réséqué

**Figure 3:**
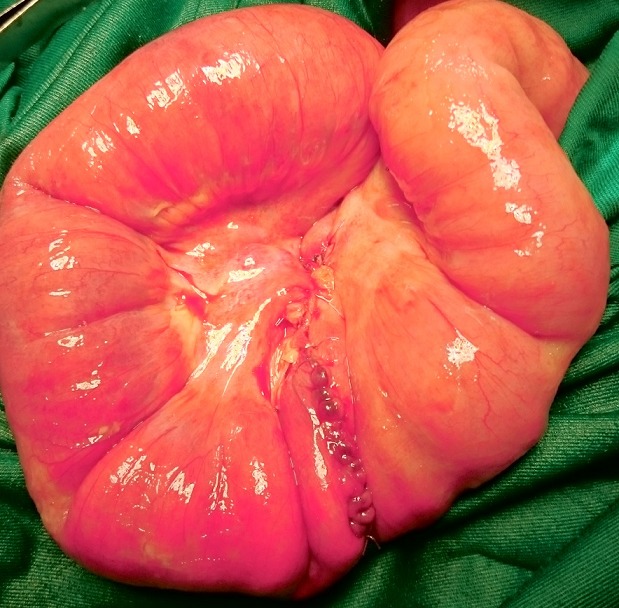
Anastomose iléo-iléale

La majorité des DM (12 cas) avait une base étroite inférieure à 2cm. Dans les cas d’occlusion (n = 10), le mécanisme était une bride tendue entre le DM et la paroi abdominale (7 cas /10) ou le mésentère (3 cas/10). Dix patients présentaient une nécrose intestinale avec perforation au moment de l’intervention (Figure 1). Cette nécrose était due à une surinfection d’une diverticulite. Une résection segmentaire du grêle emportant le diverticule était réalisée chez tous les patients (Figure 2). Cette résection était suivie d’une anastomose immédiate dans 12 cas (Figure 3). Trois patients avaient eu une iléostomie suivie d’un rétablissement de la continuité aux 30^ème^, 36^ème^ et 40^ème^ post opératoires. Deux cas de péritonites étaient notés à J4 et J5 post-opératoires. Un décès était survenu dans un tableau de choc septique chez une patiente de 70 ans. Deux patients avaient présenté une fistule digestive dont l’évolution spontanée était favorable. Sur le plan anatomopathologique, tous les DM siégeaient sur le grêle distal (dernier 1/3). Le tissu de raccordement du diverticule était fibreux et avasculaire dans tous les cas. A l’histologie, 3 cas d’hétérotopie muqueuse ont été constatés dont une hétérotopie gastrique, une colique et une association d’hétérotopies colique et gastrique chez le même patient.

## Discussion

Le diverticule de Meckel dans notre étude est une pathologie de l’adulte jeune de sexe masculin. Ces données sont conformes à celles de la littérature [1, 2]. Ainsi Park aux USA sur un effectif de 1.476 patients trouvait un sex-ratio de 3 et un âge moyen de 27 ans. Les complications intéressent 4,1 à 16% des DM. Plusieurs facteurs sont associés à ces complications. Il s’agit de l’âge jeune inférieur à 50 ans, le sexe masculin, une base diverticulaire de diamètre inférieur à 2cm et la présence de tissu ectopique [1-3]. Tous ces paramètres sont vérifiés dans notre série comme dans celle de Park qui comptait 1.476 patients. L’occlusion intestinale et l’irritation péritonéale sont les 2 principales circonstances de découverte. Elles représentent 26,2 et 55% des complications selon l’étude de Mendelson [3]. Les mécanismes de l’occlusion sont multiples par bride, inflammation, invagination ou tumeur. Ceux de la péritonite sont la nécrose, la perforation et la diffusion d’une diverticulite. Dans notre série, une bride était toujours impliquée dans l’occlusion et une nécrose intestinale avec perforation était retrouvée dans les cas de péritonite.

Le diagnostic se fait habituellement en per opératoire pour une indication non exclusivement guidée par la suspicion de DM. Le diagnostic du DM par l’imagerie fait classiquement appel à l’opacification du grêle (de préférence par entéroclyse), à la scintigraphie au ^99m^ Tc pertechnetate [4]. L’angiographie peut confirmer le diagnostic dans les formes hémorragiques [5]. Le cliché radiographique d’abdomen sans préparation et l’échographie sont peu utiles dans le diagnostic de DM. La distinction au scanner entre une anse intestinale et le diverticule de Meckel reste difficile [6]. Rarement l’existence d’un DM est suspectée en pré-opératoire. Chez un de nos patients, le scanner avait permis la suspicion d’un diverticule avant l’intervention. Mais en définitive, seule l’indication opératoire compte, l’exploration chirurgicale faisant le diagnostic [7].

La prise en charge des DM non compliqués de découverte fortuite est sujette à controverse. Par contre, devant une complication, la résection segmentaire emportant le diverticule est la règle. Elle est préférée à la résection cunéiforme dont le risque est de laisser du tissu ectopique en place [7]. La coelioscopie occupe une place de plus en plus importante dans la réalisation de ces gestes. Quelle que soit la voie d’abord utilisée, les sutures digestives doivent porter sur une zone parfaitement saine, non inflammatoire et bien vascularisée [8]. La prise en charge des DM compliqués est basée sur une réanimation initiale suivie d´une antibiothérapie et d’une résection segmentaire emportant le diverticule avec toilette péritonéale en cas de péritonite [9]. Le rétablissement de la continuité digestive peut être fait soit immédiatement par une anastomose termino-terminale soit différé avec une dérivation temporaire. Dans notre série, tous les patients ont bénéficié d’une résection segmentaire dont 12 avec anastomose immédiate.

La chirurgie des DM n’est pas exempte de complications. Nous avons noté un décès et 5 cas de complications post opératoires soit une mortalité de 6% et une morbidité de 33%. L’équipe de la Mayo Clinic rapporte une mortalité de 1% pour une morbidité de 13%. Mendelson situe cette mortalité entre 5 et 10% pour les DM compliqués. L’existence d’une hétérotopie muqueuse est fortement corrélée à la survenue d’une complication. Ainsi Park dans son étude incluant 1.476 patients retrouve un taux de 43,4% d’ectopie muqueuse sur des DM compliqués contre un taux de 14,2% de DM non compliqué. Nous avons retrouvé 3 cas d’hétérotopie muqueuse. L’hétérotopie gastrique est la plus fréquente. Plusieurs auteurs comme Williams ont retrouvé de nombreuses hétérotopies muqueuses (gastrique, pancréatique, duodénal, biliaire et colique) [10]. Il faut noter la rareté de l’hétérotopie colique qui était présente chez un de nos patients.

## Conclusion

Le diagnostic de DM compliqué se fait surtout en per-opératoire. Il s’agit d’une urgence chirurgicale nécessitant une prise en charge précoce et adéquate. Cependant la morbidité et la mortalité ne sont pas négligeables.

### Etat des connaissances actuelles sur le sujet

- Le diverticule de Meckel (DM) est une malformation rare;
- Généralement bénin et asymptomatique, il est surtout découvert fortuitement;
- Sa découverte se fait souvent à l’occasion de complications.

### Contribution de notre étude à la connaissance

- Décrire les aspects épidémiologiques, cliniques, anatomopathologiques et thérapeutiques des complications des DM sur une série de 15 cas;
- Comprendre l’implication des DM dans certaines occlusions intestinales.

## Conflits d’intérêts

Les auteurs ne déclarent aucun conflit d'intérêts.
